# Protocol for intraventricular injection of retinoids and hypothalamic application of viral vectors in the rat and mouse brain

**DOI:** 10.1016/j.xpro.2025.104329

**Published:** 2026-01-31

**Authors:** Peter I. Imoesi, Cristian M. Olarte-Sánchez, Lora Heisler, Peter McCaffery

**Affiliations:** 1 School of Medicine, University of St Andrews, St Andrews, Scotland KY16 9TF, UK; 2 Rowett Institute, School of Medicine, Medical Sciences and Nutrition, University of Aberdeen, Foresterhill, Aberdeen, Scotland AB25 2ZD, UK; 3 Institute of Medical Sciences, University of Aberdeen, Foresterhill, Aberdeen, Scotland AB25 2ZD, UK

**Keywords:** Health sciences, Model organisms, Gene expression, Neuroscience

## Abstract

The brain, specifically the hypothalamus, is a key regulator of the homeostatic balance of numerous biomolecules in the body via its control of the neuroendocrine system. Here, we present a protocol to study body vitamin A balance by injecting synthetic retinoids into the third ventricle of the rat brain. We also describe steps for the intraventricular application of viral vectors to the arcuate nucleus of a mouse brain to knock down vitamin A homeostatic genes. This approach is applicable to the study of vitamin A homeostasis.

For complete details on the use and execution of this protocol, please refer to Imoesi et al.^1^

## Highlights

• Protocol to study vitamin A homeostasis in the rodent body
• Steps for injecting synthetic retinoids into the third ventricle of the rat brain
• Procedures for the intraventricular application of viral vectors

## Abstract (editor highlights)

Publisher’s note: Undertaking any experimental protocol requires adherence to local institutional guidelines for laboratory safety and ethics.

## Graphical abstract

**Figure:**
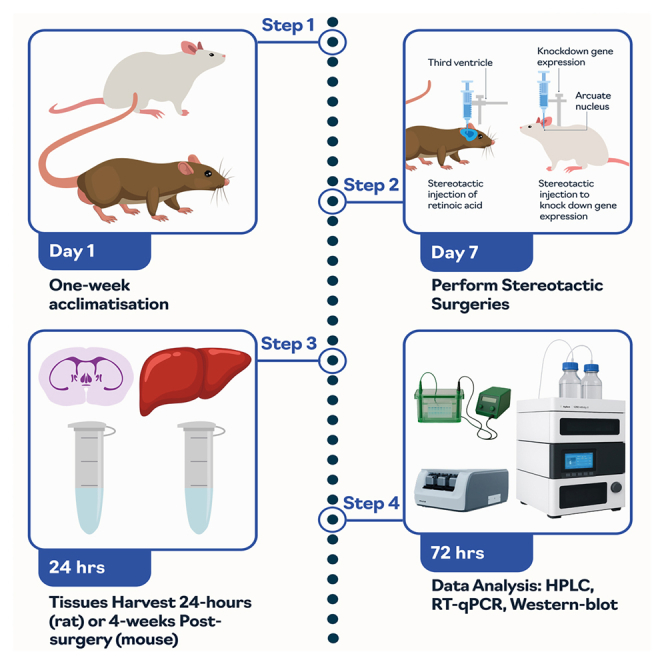

## Before you begin

Vitamin A is an essential lipid soluble micronutrient required throughout life.^2^ It is stored in the form of retinyl palmitate and released in the circulation as retinol (ROL). Circulatory ROL is maintained at a concentration of 1–3 μM, depending on species, and this concentration is maintained both in vitamin A dietary deficiency or excess intake of vitamin A,^3^ and decline in circulatory ROL concentration only occurs with chronic vitamin A deficiency.^1^ However, how this plasma concentration is maintained under most dietary circumstances is poorly understood. In 2023 we demonstrated for the first time, through the application of ROL or retinoic acid (RA) to the third ventricle of the rat brain, that the hypothalamus can regulate vitamin A homeostasis.^1^ In the same publication, using the same protocol and a set of coordinates targeted at the arcuate (ARC) nucleus of the mouse brain, we demonstrated that prolonged disruption of a vitamin A homeostatic gene i.e., the knockdown of retinol binding protein 4 (*Rbp4*) through the stereotactic application of adeno-associated virus (AAV) shRNA containing construct in the hypothalamic ARC nucleus of the mouse brain, altered ROL levels in the liver.

### Innovation

The stereotactic injection of synthetic retinoid into the third ventricle of a rat brain is technically challenging in part due to surgical and substance complications. The surgical difficulty is due to the complexity of targeting the midline region of the brain i.e., the third ventricle which is small and challenging to target compared to other regions like the hippocampus. One of such complexities is establishing the right coordinates and the angling of the injection to avoid sagittal sinus rupture. Further, wrong injection volume of synthetic retinoid or incorrect rate of injection may lead to increased intracranial pressure or direct brain damage.^4^ Unlike standard stereotactic procedures that target larger or lateral brain regions, the steps provided in this protocol allow the precise targeting of the third ventricle. This approach establishes a connection between hypothalamic activity and peripheral vitamin A metabolism, providing a unique approach for studying micronutrient regulation and neuroendocrine control.

### Animals

Eight-week-old Sprague Dawley male rats weighing 250–400 g, and 10–12-weeks-old C57BL/6 adult female mice weighing 20–25 g, were used in this protocol. The male rats were commercially obtained from Envigo RMS, Inc., and the mice obtained from the breeding wing of the Medical Research Facility–University of Aberdeen. Rats or mice were allowed access to food (CRM (P) Rat and Mouse Breeder and Grower, standard pelleted diet, Special Diet Services) and tap-water *ad libitum*. All experimental animals were approved by the ethics committee of the University of Aberdeen, in accordance with the UK Home Office guidelines, licensed under the Animals (Scientific Procedures) Act, 1986.

***Note:*** Before this protocol is applied, ethical approval should be obtained from relevant institution(s).

### Preparation of stock solutions of retinoids

**Timing: 30 min**

To obtain a solution of final concentration of RA of 0.1 molar (M).

1. Dissolve 0.3 g (300 mg) of RA in 10 mL dimethyl sulfoxide (DMSO; Sigma Aldrich) in a dark room with a low intensity yellow light source to avoid isomerization.
2. Aliquot the solution into 1.5 mL microtubes, and gently blow nitrogen gas into the tube to displace the presence of oxygen that may result in oxidative degradation of RA.
3. Wrap the microtubes with aluminum foil to prevent direct light contact and store in a –70°C freezer for subsequent use.

For viral construct (AAV9-GFP-U6-m-RBP4-shRNA, 3.5 × 10^13^ GC/mL) and the scrambled construct (AAV9-GFP-U6-m-scrmb-shRNA, 4.6 × 10^13^ GC/mL).

4. Aliquot into 0.6 mL microtubes and store in a - 70° C freezer.

### Preparation for surgery

5. Autoclave sterilization of surgical materials and tools.
  a. Use a medium size Granton Self Seal Sterilisation Pouch (GSSSP) to hold surgical materials and tools.
  b. Place into the pouch one each of:
    i. Fine scissors (sharp), suture tying forceps, mayo-hegar needle holders,
    ii. 10–15 absorption triangles (Unmounted from Fine Science Tools, Inc).
    iii. 10–15 cotton swab sticks, a drill bit, and a pencil.
    iv. 5–6 large square pieces of aluminum foil.
    v. Seal off the pouch for autoclaving.

Use two separate large GSSSPs to autoclave clean and re-usable surgical gowns and re-usable drapes. Seal off the pouch for autoclaving.

***Note:*** It is highly recommended to use one set of autoclaved materials tools for each individual animal. If a total of five surgeries is scheduled per day, five sets of autoclaved surgical materials and tools should be prepared in advance. This is important to avoid cross-contamination between animals during surgeries and avoid post-surgery infection.

**Figure 1:**
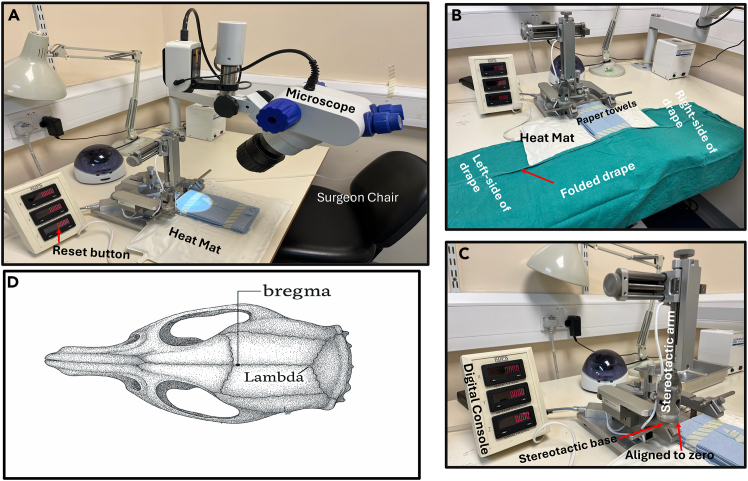
Surgical setup (A) Setup the stereotactic frame to ensure the AP, ML and DV reads on the digital console. Turn on the heat mat, microscope and adjust the surgeon chair for right height and comfort. (B) Place a sterile surgical drape underneath the stereotactic frame and make a one-step fold of the drape. Keep the drape folded until the animal is fully secure on the frame and before the start of surgery unfold the drape. (C) Ensure the stereotactic arm aligns at point zero to the stereotactic base. (D) The skull of a rat showing bregma and lambda positions.

***Note:*** Coordinates should be verified and validated with a pilot animal using a dye.

6. Animal preparation.
  a. Group house animals under constant environmental conditions (20–22°C temperature and 50–65% humidity) with light cycle kept on 12-h light/dark (lights on 7 am). Animals not bred within the facility are allowed one-week acclimatization before surgery.
  b. Record each animal’s general well-being, weight, ID, sex, genotype, the name of surgeon, and date of surgery using a record sheet.
  c. Prepare new home cages (with standard enrichment beddings) for the animal post-surgery.
    i. Mash standard pelleted diet in a polystyrene bag using warm tap-water. and.
    ii. Post-surgery, place a portion of the mashed diet in a petri-dish cover and place the diet inside the home cages with free access to water.
      ***Note:*** Standard animal diet should be made available above the cage.
  d. Set the heating cabinet at approximately 35°C and verify temperature with an independent thermometer probe before placing cages in the heat cabinet, or onto the heat-mat covered with drapes.
7. Plan the craniotomy using set coordinates.
  a. Validate the coordinates to reach the target brain region of interest using a rat brain (third ventricle AP: −0.8 mm, ML: 0.00 mm, DV: −5.2 mm from bregma, Figure 1D) or the ARC nucleus of a mouse brain (AP: −1.43 mm, ML: ±0.25 mm, DV: −5.8 mm).^5^^,^^6^ The set coordinates can be further validated using an online atlas–labs.gaidi.ca/rat-brain-atlas/or labs.gaidi.ca/mouse-brain-atlas/.

8. Preparation of surgery station.
  a. Check all surgical tools are autoclaved (using the standard autoclave procedure of the respective institution), dried and cool before use. Sterile surgical tools should be kept within the surgeon’s reach in the surgical area.
  b. Tools should include a one-off sterile and disposable safety scalpel.
  c. Check there is sufficient isoflurane, and oxygen in the cylinder.
  d. Pre-fill a 5 mL syringe with sterile saline, keep on a heat-mat and covered with a drape to pre-warm. To be used for rehydration of the animal.
  e. Pre-fill a 5 mL syringe with sterile saline and insert in a 100 mL beaker with wet ice to cool. To be used to minimize bleeding during procedure.
  f. Prepare solutions (see materials and equipment).
  g. Clean the stereotactic frame and the actual surgical area with 70% ethanol Figure 1A.
  h. The Technical Surgical Assistant (TSA) should open the pre-autoclaved drape from the GSSSP and hold the drape on both edges.
    i. The TSA should raise the stereotactic frame and underneath the frame the surgeon should carefully tuck in the sterilized drape.
    ii. Fold the drape once Figure 1B on the base of the stereotactic frame to keep the inner drape sterile–Figure 1B.
9. Preparation of the clipping and anesthesia workstation.
  a. Disinfect the workstation (Figures 2A and 2B) with 70% ethanol.

**Figure 2:**
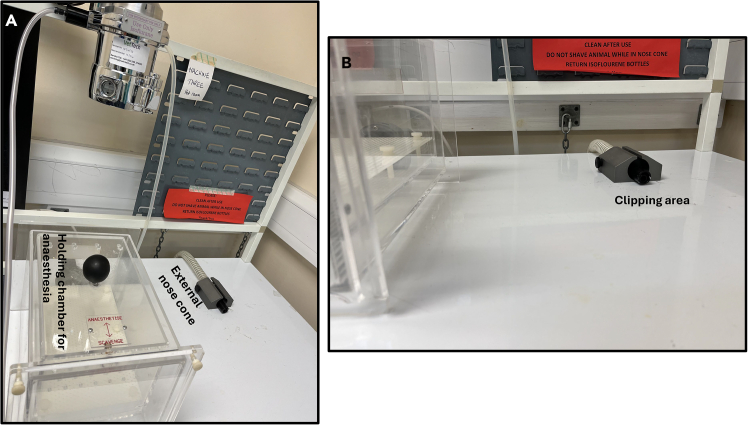
Anesthesia and clipping workstation (A) Animal holding chamber for anesthesia. Place the animal into the box and gently release isoflurane into the box. Once the animal is anesthetized, move the animal to the external nose cone and gently release the isoflurane again. (B) Whilst the animal is on the external nose cone, press the paw of the animal for reflex, no reflex indicates the animal is under anesthesia. Next, proceed to clip the surgical area.
  b. Put the cordless hair trimmer (commercially available–Wahl cordless trimmer) to charge and disinfect with 70% ethanol before use.
  c. Turn-on the heat-mat in the workstation and layer the mat with standard paper towels.
  d. Pre-prepare nonsterile cotton wool and fill a 100 mL beaker with warm tap-water.
  e. Disinfect the rat or mouse anesthetic holding chamber (box) Figure 2A by wiping with 70% ethanol and allow a bit of time for the ethanol to dispel.
  f. Place the animal into the holding chamber (box) and anesthetize with isoflurane 0.8–1.2 L/min in 2% oxygen (or follow institutional specifications).
    ***Note:*** Maintain close monitoring, look into the chamber constantly to observe when the animal is slowly induced with isoflurane. After 1–2 min in the chamber press the paw of the animal to check for reflexes or whisker movement. The absence of reflex or 40–50 breaths per minute indicates a good depth of anesthesia.
  g. Turn off the isoflurane in the chamber and switch to an external rat or mouse nose cone Figure 2A.
    i. Rest the nose cone on the pre-heated heat-mat previously layered with paper towels.
    ii. Place the animal on the heat-mat and tuck the tail underneath the stomach to reduce hypothermia.
  h. When the animal is moved to the external nose cone, allow for 1–2 min to ensure the animal is still anesthetized before clipping the surgical area (head).
    ***Note:*** At the end of clipping, slightly moisten the nonsterile cotton wool using the warm tap-water in the 100 mL beaker and clean out the fur from the surgical area.
    i. Inject 5 mg/kg (0.1 mL/100 g s.c.) of Rimadyl® Small Animal Solution (Rimadyl™, Zoetis) subcutaneously at least 10 min before surgery.
  i. Also inject 2.5 mL each of pre-warm sterile saline subcutaneously to the left and right side of the animal for rat, and 1.5 mL for mouse.
    ***Note:*** This is to keep the animal hydrated throughout the surgery.
10. Transference of the animal to the stereotactic frame–surgical area.
  a. Switch on the isoflurane to the nose cone on the stereotactic frame with 1–5% oxygen before transferring the animal onto the frame. Regulate the isoflurane within the same range during surgery.
    ***Note:*** For mouse surgery, regulate isoflurane between 1–2% oxygen.
  b. Secure the animal on the appropriate incisor bar and fit the nose cone; allow for 1- 2 min for the animal to remain anesthetized and become stabilized.
    i. Check the animal is still anesthetized.
    ii. Further secure the animal using both the left and right ear-bars ensuring both read equally in millimeter (mm) and the animal’s head is not tilted down or upward i.e., the head is properly secured horizontally.
      **CRITICAL:** To ensure the head is horizontally positioned, set lambda and check flatness of the skull: if the difference between bregma and lambda (Z-axis) has a negative value the nose is tilting upward. If the difference between bregma and lambda (Z-axis) has a positive value, the nose is tilting downward. The difference between bregma and lambda (value of Z) should not exceed ± 0.08. If the skull is not horizontally positioned, release the mask and readjust the incisor bar. To establish this accuracy, move the syringe from bregma to lambda and when the syringe is positioned on lambda, the difference should not exceed ± 0.08.
  c. Pre-fill the 5 μL Hamilton syringe with slightly more than the required injection volume.
    ***Note:*** Gently expel any air bubbles and set the exact volume before securing the syringe in the stereotactic needle-holder.
  d. Secure the syringe using the stereotactic syringe holder and rest the animal on the heat-mat attached to the stereotactic baseplate.
    ***Note:*** Tuck the tail beneath the abdomen to reduce heat loss and cover the non-surgical area with a transplant sterile nylon sheet to allow continuous observation of respiration.
  e. Disinfect the clipped scalp area with Vetasept® povidine-iodine antiseptic solution (XHG012; Animalcare) or alternatively with 70% ethanol (avoid 100% ethanol due to skin irritation). Apply Viscotears® liquid gel to both eyes to prevent corneal drying during anesthesia.
11. Preparation of surgeon in gowning area.
  a. Disinfect the gowning area and table and place a large sterile autoclave drape on the table.
  b. Open the GSSSP with the surgical gown and place on the large drape on the table.
  c. Open the sterile surgical gloves and place onto the large drape on the table.
  d. Open and put on a Disposable Nose Mask-3M.
  e. Put on a standard disposable hair covering.
  f. Proceed to a water-tap and wash hands with soap in running water. Next, dry hands with clean paper towels and scrub hands with commercially available 99.99% Hand Gel. Wait a few minutes until hands are properly dry.
  g. Gently insert hands through the surgical gown inside-out, avoiding contact with the outer part of the gown. Request the help of the TSA to help knot the gown behind.
  h. Proceed to put on the sterile surgical gloves. From this point, ensure only to handle sterilized tools throughout the surgical procedure.

**CRITICAL:** Once the surgeon is fully gowned up, only the TSA/anesthetist should open the GSSSP pouches containing sterilized instruments and disposable scalpel. The TSA presents each instrument aseptically and the surgeon places it onto the sterile drape under the stereotactic frame (extending laterally for easy access; see Figure 1B). The TSA/anesthetist monitors and regulates isoflurane and oxygen flow, while the surgeon observes the animal’s breathing rate and communicates adjustments as needed. Clear communication between the surgeon and the TSA/anesthetist is essential to maintain an appropriate anesthetic depth and ensure animal safety throughout the surgery.

## Key resources table

**Table:** 

REAGENT or RESOURCE	SOURCE	IDENTIFIER
**Bacterial and virus strains**		

AAV9-GFP-U6-m-RBP4-shRNA	Vector Biolabs	Lot# 171120#32
AAV9-GFP-U6-scrmb-shRNA	Vector Biolabs	Lot# 170814-171201

**Chemicals, peptides, and recombinant proteins**		

Isoflurane	IsoFlo®, Zoetis	N/A
Rimadyl® Small Animal Solution	Rimadyl™, Zoetis	N/A
All-trans-Retinoic Acid	Sigma-Aldrich	Cas# 302-79-4; SKU: R2625-100MG
Ethanol	Fisher Scientific	Cas# 64-17-5
Retinol	Sigma-Aldrich	Cas# 68-26-8; SKU: R7632-100MG
Vetasept® Povidine-Iodine Antiseptic Solution	Animalcare	XHG012
Dimethyl sulfoxide	Sigma Aldrich	Cas# 67-68-5

**Experimental models: Organisms/strains**		

Female Mouse: C57BL/6	Medical Research Facility, University of Aberdeen	N/A
Rat: Sprague Dawley Male Rats	Envigo RMS, Inc	N/A

**Other**		

Fine Scissors	Fine Science Tools	Item No: 14060-09
Suture Tying Forceps	Fine Science Tools	Item No: 18025-10
Mayo-Hegar Needle Holders	Fine Science Tools	Item No: 12004-16
Absorption Triangles–Unmounted	Fine Science Tools	Item No: 18105-04
Model 942 Small Animal Stereotaxic Instrument with Digital Display Console	Kopf® David Kopf Instruments	N/A
Viscotears® Liquid Gel	Commercially	N/A
Hand Gels (99.99%)	Commercially	N/A
Bone-Wax Surgical Haemostatic	Smi, Belgium	REF# Z046
Non-absorbable Suture 5.0 Ethilon™ Polyamide 6	MEDI-Move	REF# W1616T
6.0 Ethicon® coated Absorbable Suture Polyglactin 910	Ethicon® Vincryl™	REF# W9500T
1.5 mL Microtubes	Greiner Bio-one	Cat# 616201
0.6 mL Microtubes	Fisher Scientific	Cat# 14-222-143
Hamilton Microliter™	Hamilton	REF #80300
Disposable Safety Scalpels	Fine Science Tools	Item No: 10000-10

## Materials and equipment

**Table:** Solutions requiring preparation before surgery

Reagent	Final concentration	Recommended dose	Amount
Carprofen (Rimadyl)	For [rat]; to prepare an injectable dose of 50 mg/mL; add 0.1 mL Carprofen to 0.9 mL sterile saline. This dilution gives a final concentration of 5mg/mL. For [mouse]; to prepare an injectable dose of 50 mg/mL; add 0.1 mL of Carprofen to 0.9 mL sterile saline. Finally take 0.1 mL from this dilution and add to 0.4 mL sterile saline. This gives a final concentration of 1mg/mL.	5 mg/kg	Based on body weight.
Retinoic acid (RA)	10^–4^ M; from 0.1M stock take 5 μL into 495 μL of DMSO and then take 10 μL into 90 μL saline.	300 g/mol	5 μL
Dimethyl sulfoxide (DMSO)	Dissolve 10 μL into 90 μL saline	10%	5 μL
Normal saline	Dissolve 90 mg NaCl into 10 mL dH_2_O.	0.9 % NaCl	As required
Hydrogen peroxide			-

***Note:*** Store saline at 4°C prior to use; stock RA stored at −70° C and once aliquot is thawed, do not refreeze. DMSO is stored at room temperature. All injectable solutions should be brought to room temperature before injection.

**CRITICAL:** The volume of Rimadyl to be injected is subject to individual body weight of the animal.

## Step-by-step method details

### Surgery—Intraventricular injection of retinoids

**Timing: 45–60 min**

This step describes the procedure of injecting retinoids (RA or ROL) into the third ventricle of the rat brain. This is equally applicable to any region of the brain with coordinates set to reach the target brain region. The actual surgery should start when step 7 above is completed.

1. Start of surgery.
  a. Sit on a surgeon stool and ask the TSA to open the one-step folded drape outward.
    i. Ask the TSA/anesthesiologist to gently open the medium-size GSSSP containing the autoclaved surgical tools.
    ii. Ask the TSA to gently release the tools to one side of the drape (left or right–Figure 1B) without touching the tools.
      ***Note:*** The side with the sterilized tools should be differentiated from the side with used surgical tools during surgery.
  b. Take out the autoclaved aluminum foils from the kit and fold around the knobs on the stereotactic frame, the microscope knobs and the drilling machine when ready for use.
  c. Part of the autoclaved tool kits is a cotton wool stick.
    i. Use one of the sticks and gently pull back the head-skin of the animal.
    ii. Use the disposable scalpel pre-opened by the TSA, cut through the head-skin in a single straight line. Place the scalpel on the side of the drape for used surgical tools.
  d. After incision, ask the TSA to slowly apply the cold sterile saline from the wet-ice to the incision site and allow for 30–60 sec to prevent any bleeding.
    i. Insert one autoclaved cotton wool bud into hydrogen peroxide; avoid over soaking the cotton wool bud.
    ii. Clean the incised surgical area, the skull, for proper exposure of bregma.
  e. Adjust the medial-lateral (ML), anterior-posterior (AP) and dorsal-ventral (DV) knobs of the stereotactic instrument ensuring the Hamilton syringe is on bregma (Figure 1D).
    ***Note:*** If a digital console is connected to the stereotactic instrument, the TSA should help zero out the console by pressing the reset (RST) button and the ML, AP, and DV will reset to 0.00, Figures 2A and 2C.
    ***Note:*** In the absence of a digital console, manually take down the reading for ML, AP, and DV for initial coordinates and workout the final coordinates using the pre-defined coordinates set to reach the target brain region. This protocol we injected into the third ventricle of the rat brain using the respective coordinates; AP–0.8 mm, ML–0.0 mm and DV–6.5 mm.
  f. Slightly raise the DV knob and take the actual coordinate for the specific brain region of interest. Once the set coordinates are determined, mark the spot with a pencil.
  g. Insert the autoclaved drill bit into the drill machine with help from the TSA.
    i. The TSA holds the drill machine in place, and the surgeon inserts the drill bit and the TSA locks in the bit.
    ii. Wrap the autoclaved paper towels around the drill machine. This is necessary to avoid the gowned surgeon contaminating their sterile surgical gloves.
  h. Gently drill the marked spot on the skull.
    **CRITICAL:** Carefully drill a hole through the skull and keep an eye on the dura. If bleeding, use the sterilized absorption triangles unmounted to apply minimal pressure to stop the bleeding. If the bleeding persists, with the help of the TSA, apply a few drops of cold sterile saline previously immersed in wet ice prior to surgery and allow for 1–3 min. Again, use the sterilized absorption triangle unmounted to absorb the bleed and saline, and hold in place for another 1–3 min.
  i. Lower the Hamilton syringe through the skull and into the brain. The depth of the needle is the DV measurement (mm) to reach the target brain region.
  j. Gently inject the retinoid solution slowly at a rate of 0.5 μL/30 s, over approximately 5-min total (∼5 μL). Maintain steady pressure to prevent reflux.
    **CRITICAL:** First eject a small amount of the injectable solution from the Hamilton syringe to ensure the syringe is not blocked.
  k. After injection, leave the needle in place for 5 min to permit diffusion and minimize backflow. Then withdraw the needle by 0.5 mm and pause for 60 s.
    i. Remove the syringe slowly over 2–3 min until fully withdrawn.
  l. Apply Bone-Wax Surgical Hemostatic (REF# Z046 smi, Belgium) to the drill hole. Ensure there is no bleeding.
  m. Suture the incised area with a non-absorbable suture material (e.g., a 5.0 Ethilon™ Polyamide 6 (REF# W1616T).
  n. During the final sutures, ask the anesthetist to gradually reduce the isoflurane concentration to facilitate recovery.
  o. Inject (subcutaneously) 2.5 mL of warm sterile saline into both sides of the animal (rat).
    i. Take out the animal from the stereotactic frame into the previously prepared home cage (see step 2c).
  p. Transfer the animal to the heat cabinet set at 35°C (see step 2d) and leave in the cabinet for 30–45 min. At this time (depending on the state of the animal), take the cage to the animal holding room for further post-op monitoring.
    ***Note:*** Whilst the animal is in the heat cabinet just after surgery, keep closely monitored. Be vigilant for possible signs of discomfort, e.g., hunched appearance, flattened ears and grimace, circling in cage, reduced activity, poor eating and sleepiness. Should any or some of these signs continue for a period of 1–3 h post-surgery, communicate with the facility Named Animal Care and Welfare Officer (NACWO) and administer analgesic and keep record of dosing. Similarly, monitor the body weight of the animal 24-, 48- and 72-h post-surgery and in the instance of substantial weight loss, loose stitches or infection communicate with a NACWO.

### Surgery—Hypothalamic injection of viral vectors

**Timing: 60 min**

Before surgery, ensure the mouse’s body temperature is maintained using a thermostatically controlled heating pad. Apply ophthalmic ointment to prevent corneal drying. Confirm deep anesthesia via pedal withdrawal reflex before fixation in the stereotactic frame. The steps and procedures described above for stereotactic injection into the rat brain is applicable for mouse stereotactic injection but with a slight adjustment. This slight adjustment is necessary to be sure the mouse head is correctly positioned horizontally.

2. Start of surgery.
  a. Follow the previous procedure–Surgery–intraventricular injection of retinoid steps 1a–d.
  b. Identify bregma and lambda.
    i. Position the Hamilton syringe to bregma and next move to lambda.
    ii. Lower the syringe to lambda and ensure a perfect horizontal positioning of the mouse head.
      ***Note:*** The distance (DV) at which the syringe is lowered to touch lambda should be between 0.00 mm–0.05 mm.
  c. Check the distance between bregma and lambda is between 0.00 mm–0.05 mm, if off, adjust the incisor bar up or down to achieve the horizontal positioning of the head.
  d. Confirm the horizontal positioning of the mouse head, move the syringe back to bregma and zero out the digital console by pressing the reset button for AP, ML and DV, Figure 2A.
  e. Take the coordinates set to reach the specific brain region of interest.
    i. To reach the ARC nucleus of the mouse brain, move the Hamilton syringe using the AP, ML arm of the stereotactic frame in line with predetermined coordinates.
    ii. To reach the ARC, use the following coordinates: AP–1.43 mm, ML–± 0.25 mm and DV: −5.8 mm from the skull surface (measured at bregma).
      ***Note:*** If manual calibration is used, determine DV by lowering the syringe to touch the skull and retracting 0.05 mm upward before zeroing.
  f. Applications requiring a bilateral injection, repeat the same coordinates on the other side of the mouse skull and with a pencil indicate the spot to drill on the skull.
  g. Gently drill a hole on one or both sides (as needed) of the skull based on the coordinates. If bleeding occurs, follow the same steps previously described above (see CRITICAL under step-by-step method details; Surgery–Intraventricular injection of retinoid. Critical is under Step 8 h).
  h. Calibrate the motorized Neurostar injector and program the Stereo-drive software to inject the precise volume (0.5 μL) of the viral construct over a period of 5 min.
    i. Repeat the same process for the other half of the skull (if needed).
    ii. Inject 0.5 μL per site (viral titre ∼10^13^ GC/mL, AAV serotype 2/8 or equivalent. Adjust concentration depending on experimental design.
    iii. Leave the syringe in place over a period of 5 min for each injection.
  i. After the injections, slowly withdraw the syringe as previously described above.
  j. Fill the drilled holes with bone-wax and suture the incised skin with a 6.0 Ethicon® coated Vicryl™ polyglactin 910 undyed braided absorbable suture (REF# W9500T).
  k. Inject (subcutaneously) 1.5 mL of warm sterile saline to both side of the mouse and follow step 1 (n) previously described above.

## Expected outcomes

Before starting any surgery, ensure the coordinates to reach the brain region of interest is established with reference to a rat or mouse brain atlas. For accurate stereotaxic injection, correct horizontal positioning of the rat or mouse head to the stereotactic frame is key (this requires accurate setting of the incisor, left and right ear bars). This positioning determines if the injected solution reaches the correct brain region of interest.

**Figure 3:**
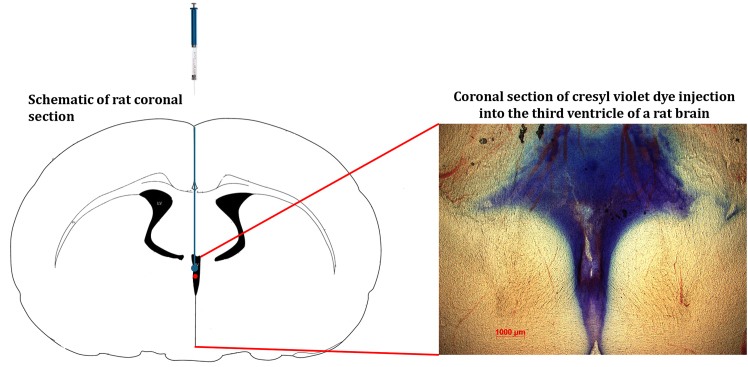
Injection of Cresyl violet dye to the third ventricle Stereotactic injection of 5 μL of cresyl violet dye into the third ventricle of a rat brain to confirm the set coordinates (AP–0.8 mm, ML–0.0 mm and DV–6.5 mm) reaches the third ventricle of the rat brain. The injection site (third ventricle) maps with the third ventricle of a coronal image from the Rat Brain in Stereotactic Coordinates, Paxinos and Watson 2007.

**Figure 4:**
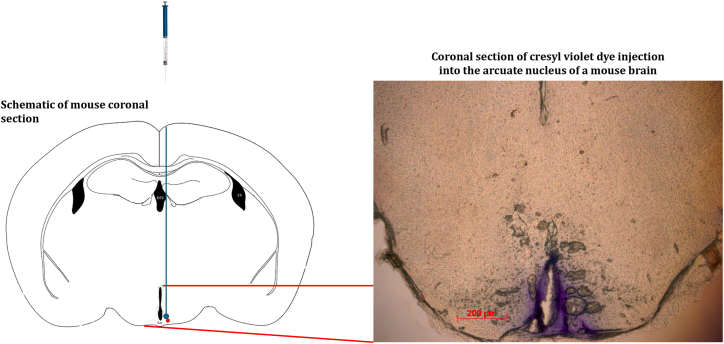
Injection of Cresyl violet dye to the arcuate nucleus Stereotactic injection of 3 μL of cresyl violet dye into the arcuate nucleus of a mouse brain to confirm the set coordinates (AP–1.43 mm, ML - ± 0.25 mm and DV–was based on the point at which the needle touches the base of the skull and then withdrawn 0.05 mm upward) reaches the arcuate nucleus of the mouse brain. The injection site (arcuate nucleus) maps with the arcuate nucleus of a coronal image from the Mouse Brain in Stereotactic Coordinates, Paxinos and Franklin, 2008.

The coordinates to reach the brain region of interest should be first tested by injecting a cresyl violet dye into a rat or mouse cadaver. In this protocol we injected a cresyl violet dye into the third ventricle of a rat brain Figure 3, and into the ARC nucleus of a mouse brain Figure 4. Once the coordinate of interest is established based on the result from the cresyl violet dye injections, the surgeon can proceed to use the said coordinates in the actual study. To determine the accuracy of retinoid injection into the third ventricle of a rat brain we measured the expression level of *Aldh1a1* mRNA transcript in the hypothalamus. The downregulation of *Aldh1a1* mRNA transcript which encodes ALDH1A1 protein serves as compensation for RA synthesis. To determine Rbp4 knockdown, we measured the expression level of *Rbp4* mRNA transcript in the hypothalamus.^1^ It is also important to include a positive control in the study to also confirm the injection reaches the target site (successful targeting of the ARC nucleus is indicated by bilaterial viral expression limited to the mediobasal hypothalamus, verified by postmortem fluorescence imaging, immunohistochemistry or the expression levels of *Rbp4* mRNA transcript.

## Limitations

The technical limitation of poor injection accuracy could be associated with the surgeon’s inexperience in small animal surgeries for instance leading to incorrect positioning of the animal on the stereotactic frame. In this protocol we used a stereotactic frame connected to a digital console which allows for accurate determination of AP, ML, and DV readings. In laboratories where coordinates readings are manually taken, care is needed for precise injection into any brain region of interest. Also, lack of straight-line drilling through the skull could guide the syringe to a different location of the brain. Hence injection of cresyl violet dye into a relevant cadaver to establish the accuracy and optimization of the surgical procedure is strongly recommended. The use of younger rats or mice instead of adult could alter the accuracy of the stereotactic coordinates which are based on adult rat or mice atlases.

## Troubleshooting

### Problem 1

During the surgical procedure for ventricular injection the needle could hit a sinus and may result in continuous bleeding.

### Potential solution

Bleeding must be stopped before continuing with the surgery. To stop the bleeding apply cold sterile saline over the site of bleeding and wait 1–3 min. Next, with sterile cotton wool, apply light pressure to the bleeding site. This could last for 3–5 min depending on the bleed. If the bleeding is continuous due to sinus damage, speak to the NACWO and consider euthanizing the animal as the last resort.

### Problem 2

Administer a pre-emptive analgesic such as meloxicam (1–2 mg/kg, s.c.) immediately after surgery, unless contraindicated. Repeat after 24 h if needed.

### Potential solution

Discomfort post-surgery may prevent the animal from reaching food hence the need to provide mashed (softened food) pellets in the first 0–3 h of surgery. If the eating and drinking difficulty persists after 3 h post-surgery, communicate with the NACWO and administer suitable painkillers. This could be subcutaneous or in the drinking water. Keep animal closely monitored for 24 h and if the discomfort persists the animal should be euthanized.

### Problem 3

Wound re-opening post-surgery. Post-surgery, small animals are known to scratch the surgical area, removing the suture material.

### Potential solution

When suturing the animal, ensure the first knot is closer to the skin and two additional knots are applied to the first knot. In the instance where the suture is removed, re-suture. Re-suturing is only allowed once and within the first 24 h post-surgery. If wound reopening occurs beyond 24 h or if infection is evident, euthanize the animal following institutional humane endpoint criteria.

## Resource availability

### Lead contact

Further information and requests for resources and reagents should be directed to and will be fulfilled by the lead contact, Peter I. Imoesi (pii1@st-andrews.ac.uk).

### Technical contact

Technical questions on executing this protocol should be directed to and will be answered by the technical contact, Peter I. Imoesi (pii1@st-andrews.ac.uk).

### Materials availability

This study did not generate new unique reagents.

### Data and code availability

This study did not generate any data and code sets.

## Acknowledgments

PJMc acknowledges funding from the UKRI Biotechnology and Biological Sciences Research Council (BBSRC) grant number BB/T00875X/1, the University of Aberdeen Elphinstone PhD Scholarship, Sir Richard Stapley Educational Trust, and the home sponsor for P.I.I. L.H. acknowledges funding from the BBSRC (BB/R01857X/1; BBV016849/1). Further support was received from BBSRC grant BB/P004806/1, and funding was also provided by an MRC Discovery award (MRC/PC/15077). Funding for publication was provided by the School of Medicine, University of St Andrews. Special thanks to Pablo B. Martinez de Morentin, Lourdes Valencia-torres, and Giuseppe D′ Agostino for their technical advice. Also, special thanks to the staff of the Medical Research Facility, University of Aberdeen, for providing the enabling environment for the surgeries and the welfare of the animals.

## Author contributions

P.I.I. led the project, designed and performed the experiments, and wrote the first manuscript draft. C.M.O.-S. performed the experiments and co-wrote the article. L.H. co-wrote the article. P.M. conceived the project, performed the experiments, and co-wrote the article.

## Declaration of interests

The authors declare no competing interests.
